# Milk production and blood metabolites of dairy cattle as influenced by thermal-humidity index

**DOI:** 10.1007/s11250-018-1513-y

**Published:** 2018-01-25

**Authors:** Thapelo W. Kekana, Florence V. Nherera-Chokuda, Mukengela C. Muya, Kabelo M. Manyama, Khoboso C. Lehloenya

**Affiliations:** 1 Livestock Nutrition Unit, Agricltural Research Council, P. Bag x 02, Irene, 0062 South Africa; 2 Faculty of Science and Agriculture, University of Zululand, KwaDlangezwa, 3886 South Africa

**Keywords:** Blood proteins, Milk production, Smallholder dairy cows

## Abstract

The effects of high thermal stress on serum protein metabolites, milk production of transition dairy cows in semi-arid areas in South Africa were evaluated. Forty, ± 8 months pregnant, Jersey heifers (± 26 months) in zero grazing management were selected during summer from two semi-arid communal areas. Summer thermal-humidity index (THI) of the areas were THI-1 (72–83: extreme caution) and THI-2 (75–87: danger). Blood samples were collected (21 days pre-partum, and 21 and 75 days post-partum) and analysed for serum protein metabolites. Milk yield was recorded daily and samples collected for milk fat, protein, lactose and urea nitrogen analysis. Heifers in THI-2 had lower (*P* < 0.05) total serum proteins, albumin and blood urea nitrogen than THI-1. Post-calving, cows in THI-1 had higher (*P* < 0.05) TP (73.4 vs 67.9 g/l) and BUN (4.61 vs 3.77 mmol/l) at 21 DIM, and lower (P creatinine at 21 and 75 DIM than THI-2 group. Milk yield, fat and protein in THI-2 were all lower (*P* < 0.05) than THI-1 21DIM. The results confirm that heat stress affects utilisation of nutrients in transition dairy cows.

## Introduction

Milk production contributes to nutrition and income security in resource poor environments of sub-Saharan Africa. However, poor animal nutrition and heat stress affect immunity and performance of dairy cows. Pregnant and lactating cows are more susceptible to metabolic disorders due to high nutrient demands for foetal growth, growth and lactation (Polsky and von Keyserlingk 2017; De Rensis et al. 2015) and increase maintenance requirements for heat dissipation (Jiminez 2013). Dairy cattle in subtropical and tropical areas are subjected to extended periods of high temperature and sometimes high relative humidity (high temperature-humidity index THI). The thermal comfort zone of dairy cows is − 13 to 25 °C (Kadzere et al. 2002; Bickert and Mattiello 2016), beyond which heat stress occurs. When rectal temperature exceeds the threshold (39.5 °C), cows pant, drool and subcutaneous blood flow increase (Garner et al. 2017). It is noted that maintenance energy requirements surge when ambient temperatures exceed 35 °C, the feed intake declines, which affect rumination and the synthesis of milk precursors (Könyves et al. 2017).

In Sub-Saharan Africa, drought and dry seasons are becoming more frequent and longer in duration due to the *El Nino* effect. The Intergovernmental Panel on Climate Change (IPCC) (2014) indicated that global temperatures would rise by 1.8–4 °C by the end of the twenty-first century. Death in cows due to hyperthermia may occur (Cox et al. 2016). High milk-producing dairy cows succumb to heat stress earlier than low milk-producing cows (Hu et al. 2016) as immunity is lowered by the stress of milk production (Tao and Dahl 2013). Management strategies including high-energy feeds, watering, shade, tunnels and sprinklers may alleviate the problem (Nickerson 2014). The effects of very high temperatures on immunity of transitioning dairy cows in semi-arid areas should be assessed as that has implications on food security of poorer communities. The objective of the study was to determine effects of high thermal stress on serum proteins and milk production of transitioning and early lactating dairy cows in semi-arid communal areas of South Africa.

## Material and methods

Forty pregnant Jersey heifers (310 ± 40-kg live weight; BCS 2.5–3.5; ± 26 months) in zero grazing management were selected from two communal areas of Limpopo province, South Africa ((site1:24.8335° S, 29.9741° E and site2:22.7696° S, 29.9741° E). Temperature-humidity indices for the period October–December 2015 were calculated from ambient temperature-AT and RH-relative humidity (ISWC, ARC; 2015/2016). The NRC (1971) equation was applied to determine THI (site1:72–83 and site2:75–87). Schüller et al. (2014) defined THI categories for dairy cattle as optimum: < 65 and danger: 75–80.

Pregnant heifers were housed in partially roofed sheds and fed 4 kg/animal/day dry cow concentrate (92.4%DM; 15.1%CP; 3.9%Fat; 41%NDF; 9.4%ADF) and postpartum-6 kg/animal/day lactating concentrate (90.4%DM; 17.3%CP; 2.7%Fat; 39.3%NDF; 7.5%ADF) with ad *adlib Eragrostis curvular* hay and clean water. Cows were hand milked (06H00) and calves suckled during the day and separated from dams overnight. Composite milk samples were collected from morning milking sessions weekly until 75 DIM and analysed for fat, CP, MUN and lactose using the System-4000-Infrared Analyser (Foss-Electric, Hillerod).

Blood was collected in anti-coagulant tubes from the coccygeal vein before concentrate feeding at 21-day pre-partum and 21 and 75 DIM. Post-blood centrifugation (1000×*g*: 10 min), the separated serum was stored pending analysis of total proteins (TP) (Doumas and Biggs 1972; Weichselbaum 1946) and creatinine (Cr) (Tietz 1995). Globulin were calculated as the difference between TP and Alb. Enzymatic methods (Tietz 1995) were used to determine blood urea (BUN). Data was analysed using analysis of variance procedures (ANOVA) for a complete randomised design (CRD) in Statistical Analysis System version 9.0 (SAS 2009). Fisher’s least significant difference (LSD) method was used to separate means and difference were declared at *P* < 0.05.

## Results

Pregnant heifers in THI-2 had lower (*P* < 0.05) TP, Alb and BUN than THI-1 (Table 1). Blood Crea and Glob pre-calving were not affected (*P* < 0.05). Lactating cows (THI-2) had higher (*P* < 0.05) TP and BUN at 21 DIM, and higher (*P* < 0.05) creatinine at + 21 and + 75 DIM than THI-1 cows. At 21 days, milk yield, fat and protein content differed (*P* < 0.05; Table 2) and no differences were noted thereafter except in MUN.

**Table 1:** Serum protein metabolites in of primiparous Jersey cows in semi-arid communal areas

Parameters	THI-1 (72–83)	THI-2 (75–87)	SEM
Pre-calving (− 21 days)			
Total protein (g/L)	72.6^b^	78.3^a^	1.53
Blood urea nitrogen (mmol/L)	4.9^b^	5.9^a^	0.28
Creatinine (μmol/L)	126	138	5.71
Albumin (g/L)	31.5^b^	39.1^a^	2.64
Globulin (mmol/L)	41.1	39.3	2.79
Post-calving			
Total protein (g/L)			
21 DIM	67.9^b^	73.4^a^	1.34
75 DIM	62.9	65.5	1.32
Blood urea nitrogen (mmol/L)			
21 DIM	3.8^b^	4.6^a^	0.21
75 DIM	4.2	4.4	0.51
Creatinine (μmol/L)			
21 DIM	97.2^b^	127^a^	7.88
75 DIM	78.9^b^	130^a^	3.28
Albumin (g/L)			
21 DIM	32.2	36.3	2.15
75 DIM	28.6	30.7	2.16
Globulin (mmol/L)			
21 DIM	35.8	37.2	2.42
75 DIM	34.4	34.8	2.64

Means with the same superscript in the same row are not significantly different at *P* < 0.05

21 and 75 = days in milk, *THI* thermo-humidity index, *SEM* standard error of mean

**Table 2:** Early lactation milk yield and composition of primiparous Jersey cows in semi-arid communal areas

Parameter	THI-1 (72–83)	THI-2 (75–87)	SEM
Mean 1–21 days in milk			
Milk yield (litres/day)	12.4^a^	10.6^b^	0.79
Fat (%)	4.5^a^	3.1^b^	0.41
Protein (%)	3.6^a^	2.6^b^	0.56
Urea nitrogen (mg/day)	13.7	11.4	1.96
Lactose (%)	4.8	3.9	0.37
Mean 22–75 days in milk			
Milk yield (litres/day)	8.6	8.4	0.79
Fat (%)	4.5	4.5	0.41
Protein (%)	3.5	3.2	0.56
Urea nitrogen (mg/day)	10.2^a^	18.3^b^	1.96
Lactose (%)	4.4	4.6	0.37

^a, b^In the same row, values with different superscripts are significantly different *P* < 0.05

*THI* temperature-humidity index, *SEM* standard error of mean

## Discussion

Although the Jersey cows were supplemented at 6 kg/cow/day, milk yield was low. Karimi et al. (2015) indicated that heat stress reduce feed intake affecting synthesis of milk precursors. During early lactation, feed intake is low and heat stress exacerbated the problem. The low milk yield from THI-2 could be associated with low albumin and globulin concentrations (Das et al. 2016), which explains indifferences in milk composition post-21 DIM as also noted (Bernabucci et al. 2014; Garner et al. 2017). Although blood TP and BUN did not differ at 75 DIM, the metabolites were low in THI-2.

Lamp et al. (2015) reported that creatinine synthesis in healthy animals is constant and depends on muscle mass and protein intake, which influence the levels in serum. High protein oxidation in pregnant animals and during early lactation compensates for low nutrient intake to support the demands for pregnancy and milk production. Proteins oxidation causes high muscle proteolysis that increases plasma urea concentrations (Greenwood and Café 2007) causing increase in creatinine. The high THI in site2 could affect kidney function and induce clearance of creatinine and circulation of urea (Higashiyama et al. 2014).

Studies by Zhang et al. (2014) and Cowley et al. (2015) confirm observations noted in this study. The imbalance in rumen protein and energy increase rumen N balance affecting plasma urea. Cow mortality in THI-2 was 9% and none in THI-1, and heat stress was implicated.

## Conclusion/recommendations

The current study revealed the negative impact of high ambient temperatures on performance of transition dairy cows especially on smallholder farms as producers only provided partial roof shed to mitigate prolonged heat. Milk production was low which would reduce sustainability of milk businesses under such high heat conditions. The goal for achieving economic and food security for the rural poor through dairy farming can only be realised through concerted research on new technologies to mitigate heat stress.
